# Supplementary figures and images for: Deciphering individual triticale grain weight patterns: A gaussian mixture model approach
Source: PLoS One. 2024 Nov 26;19(11):e0313942. doi: 10.1371/journal.pone.0313942 (PMC11594513; doi:10.1371/journal.pone.0313942)

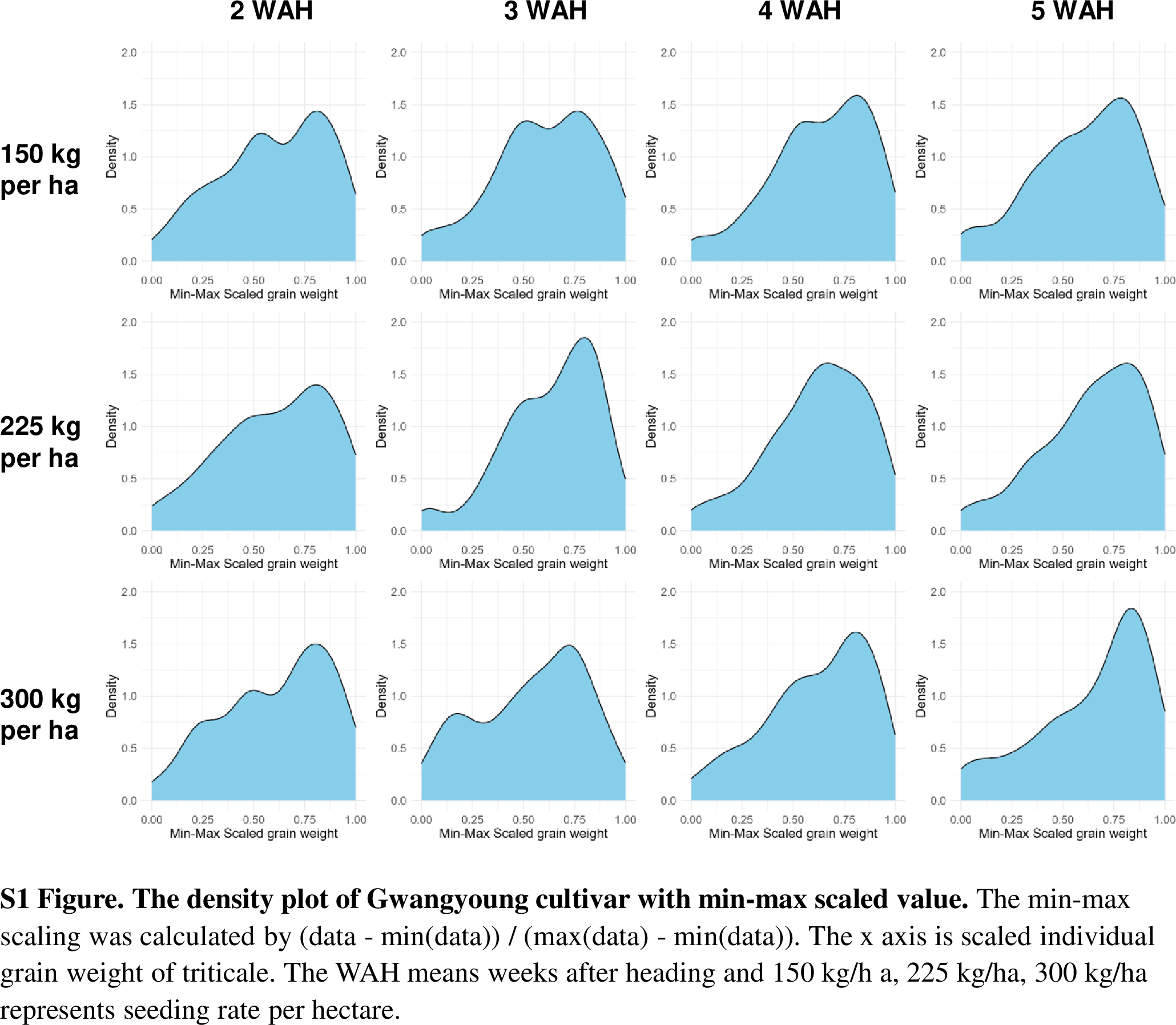

Supplement: S1 Fig — The min-max scaling was calculated by (data—min(data)) / (max(data)—min(data)). The x axis is scaled individual grain weight of triticale. The WAH means weeks after heading and 150 kg/h a, 225 kg/ha, 300 kg/ha represents seeding rate per hectare. (TIF) [file pone.0313942.s001.tif]

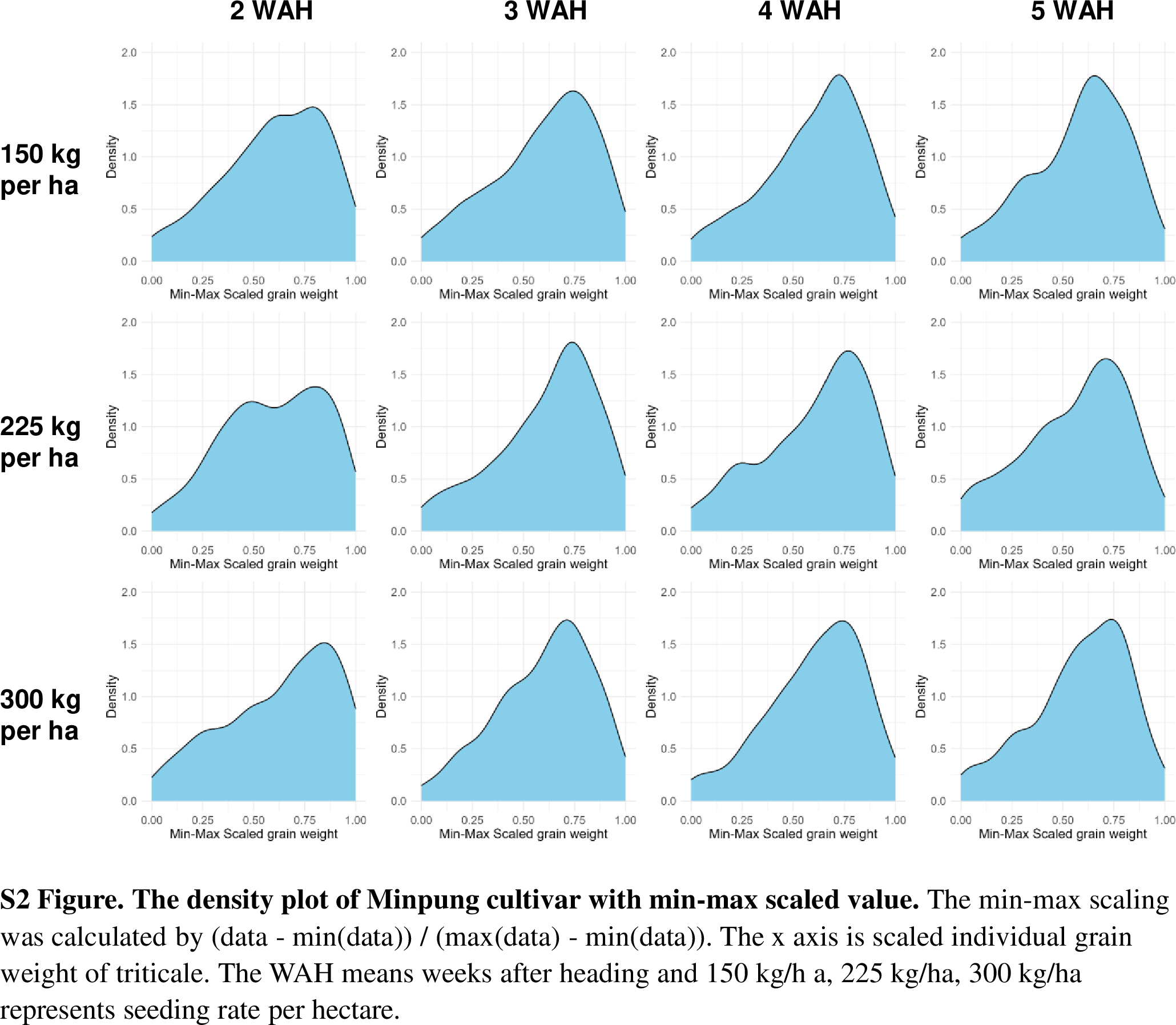

Supplement: S2 Fig — The min-max scaling was calculated by (data—min(data)) / (max(data)—min(data)). The x axis is scaled individual grain weight of triticale. The WAH means weeks after heading and 150 kg/h a, 225 kg/ha, 300 kg/ha represents seeding rate per hectare. (TIF) [file pone.0313942.s002.tif]

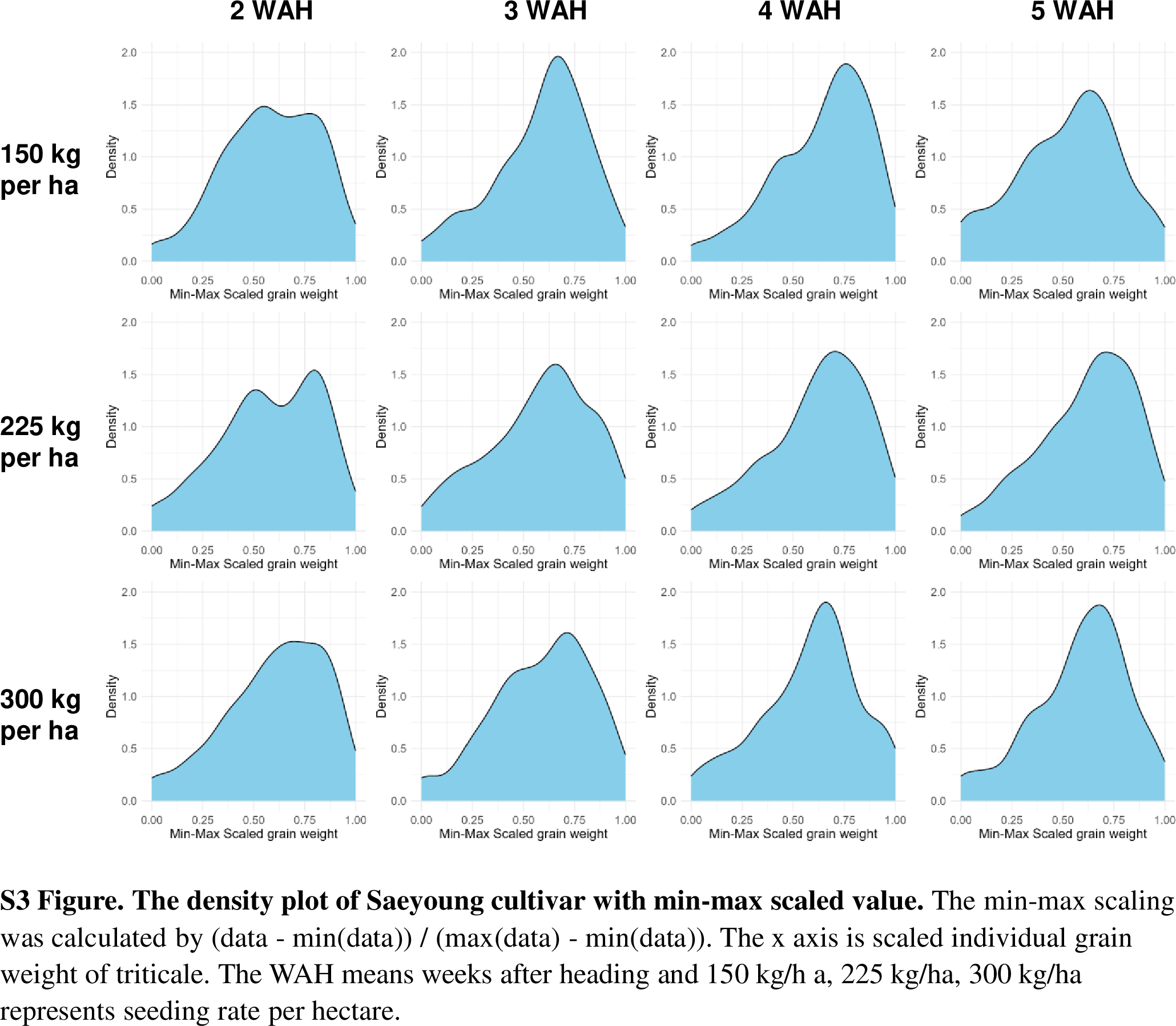

Supplement: S3 Fig — The min-max scaling was calculated by (data—min(data)) / (max(data)—min(data)). The x axis is scaled individual grain wight of triticale. The WAH means weeks after heading and 150 kg/h a, 225 kg/ha, 300 kg/ha represents seeding rate per hectare. (TIF) [file pone.0313942.s003.tif]

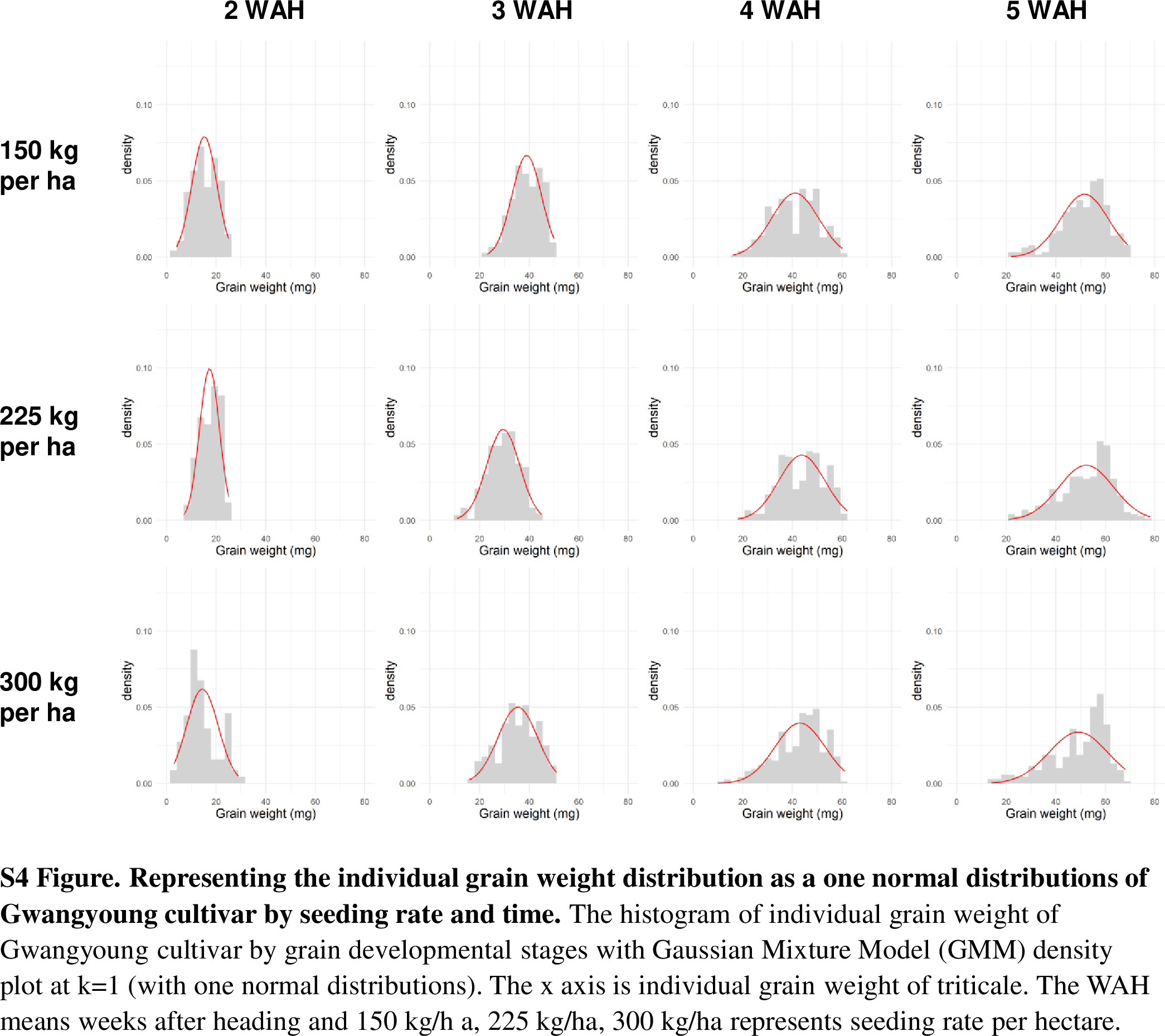

Supplement: S4 Fig — The histogram of individual grain weight of Gwangyoung cultivar by grain developmental stages with Gaussian Mixture Model (GMM) density plot at k = 1 (with one normal distributions). The x axis is individual grain weight of triticale. The WAH means weeks after heading and 150 kg/h a, 225 kg/ha, 300 kg/ha represents seeding rate per hectare. (TIF) [file pone.0313942.s004.tif]

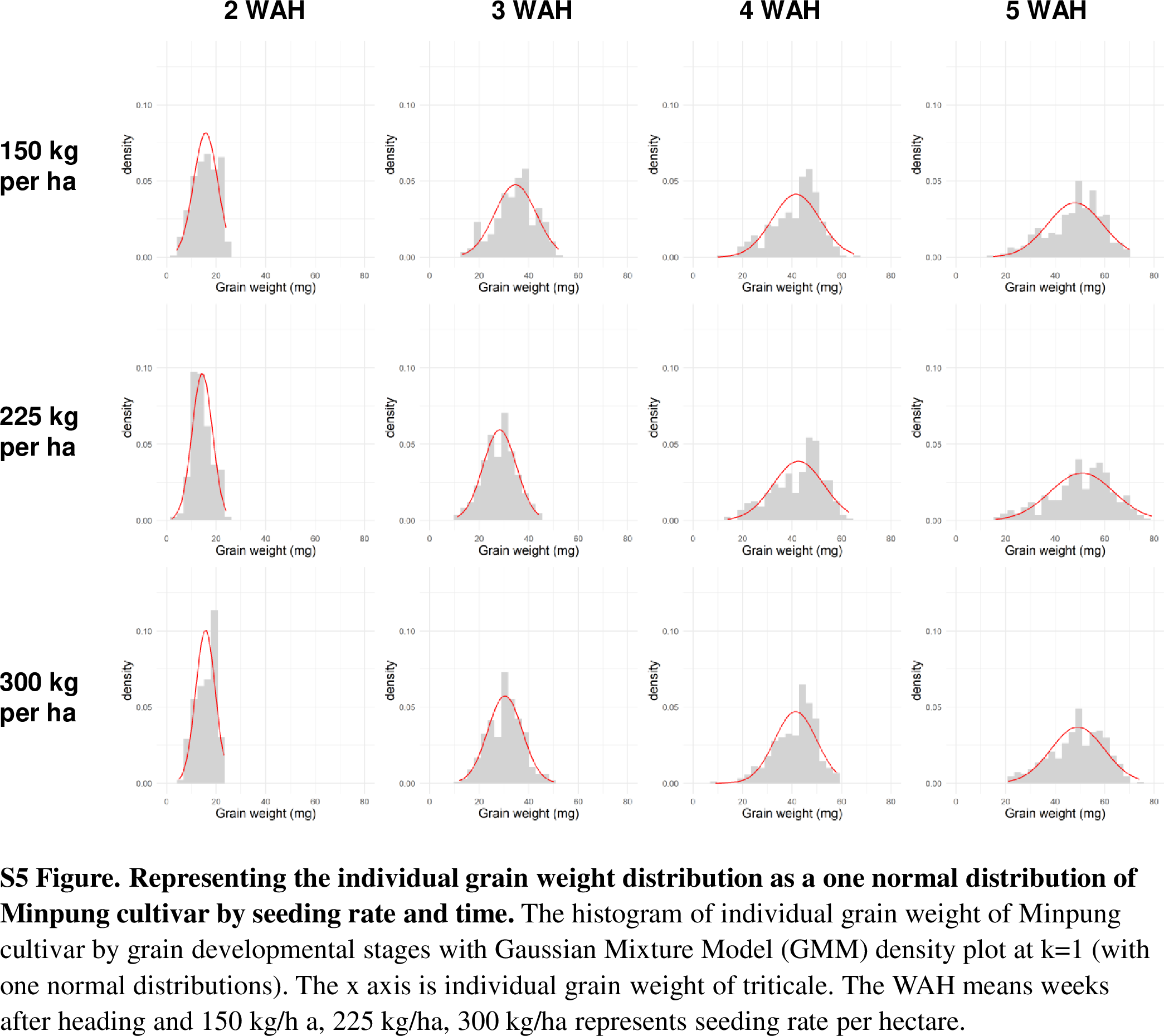

Supplement: S5 Fig — The histogram of individual grain weight of Minpung cultivar by grain developmental stages with Gaussian Mixture Model (GMM) density plot at k = 1 (with one normal distributions). The x axis is individual grain weight of triticale. The WAH means weeks after heading and 150 kg/h a, 225 kg/ha, 300 kg/ha represents seeding rate per hectare. (TIF) [file pone.0313942.s005.tif]

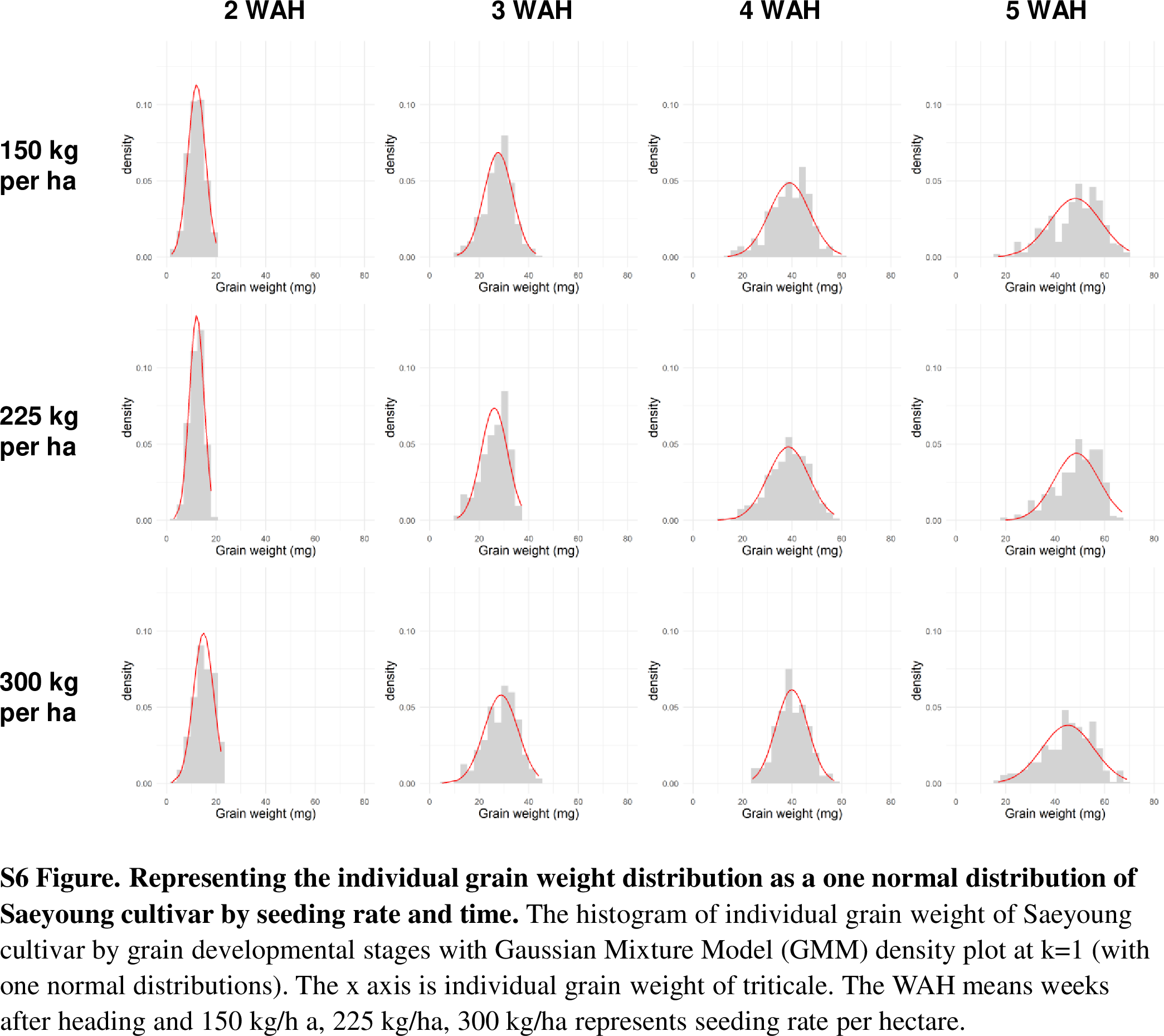

Supplement: S6 Fig — The histogram of individual grain weight of Saeyoung cultivar by grain developmental stages with Gaussian Mixture Model (GMM) density plot at k = 1 (with one normal distributions). The x axis is individual grain weight of triticale. The WAH means weeks after heading and 150 kg/h a, 225 kg/ha, 300 kg/ha represents seeding rate per hectare. (TIF) [file pone.0313942.s006.tif]

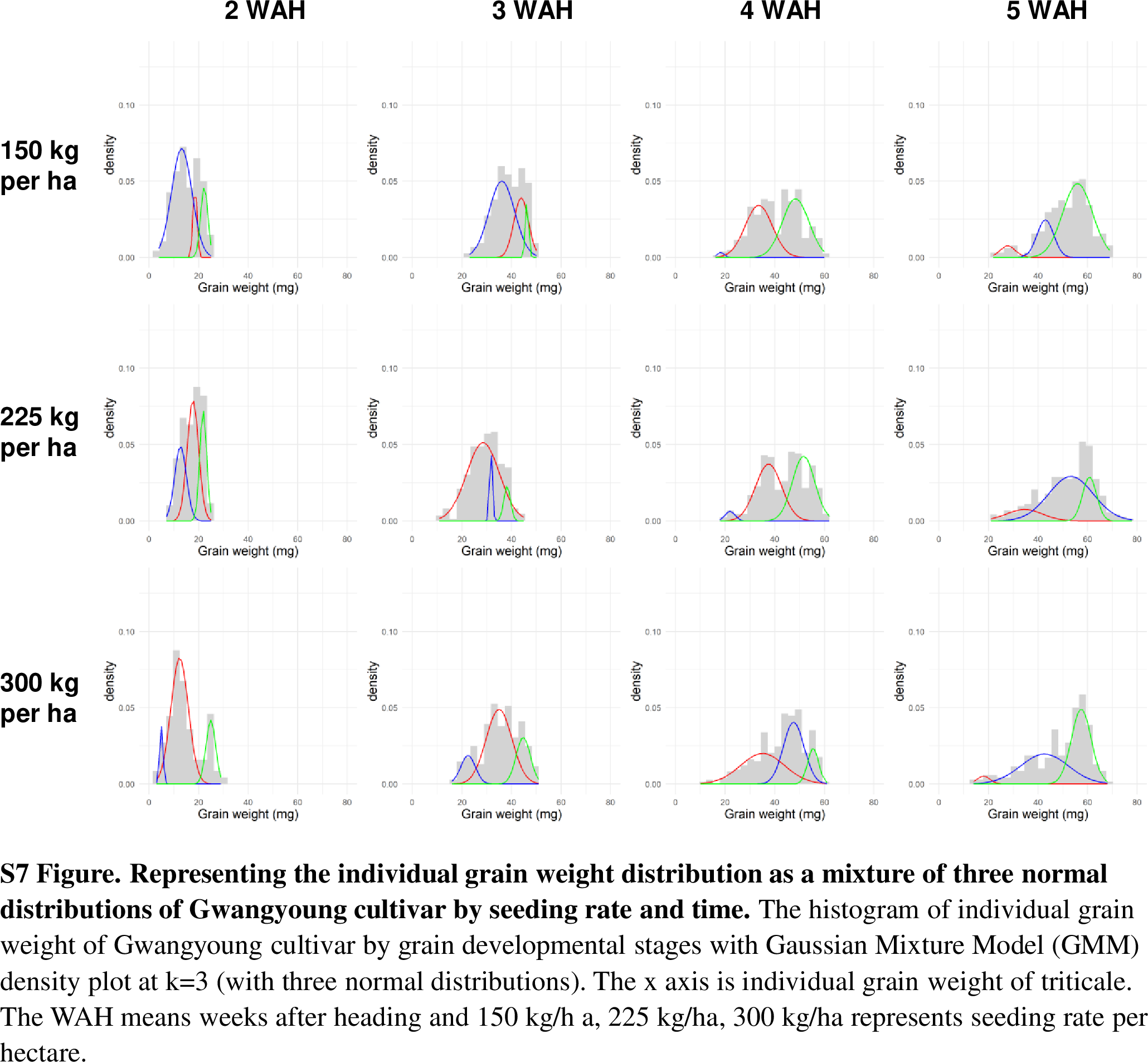

Supplement: S7 Fig — The histogram of individual grain weight of Gwangyoung cultivar by grain developmental stages with Gaussian Mixture Model (GMM) density plot at k = 3 (with three normal distributions). The x axis is individual grain weight of triticale. The WAH means weeks after heading and 150 kg/h a, 225 kg/ha, 300 kg/ha represents seeding rate per hectare. (TIF) [file pone.0313942.s007.tif]

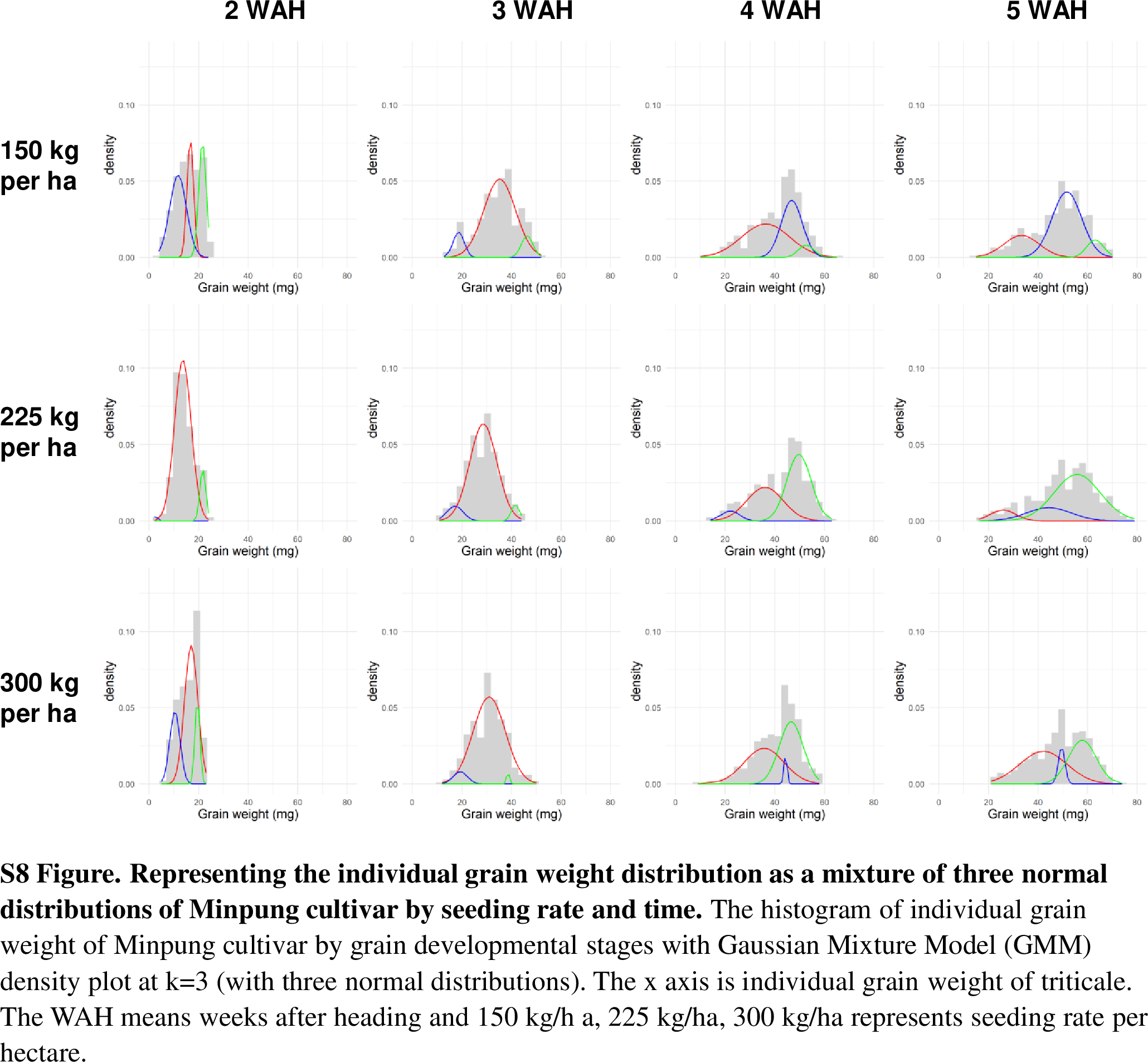

Supplement: S8 Fig — The histogram of individual grain weight of Minpung cultivar by grain developmental stages with Gaussian Mixture Model (GMM) density plot at k = 3 (with three normal distributions). The x axis is individual grain weight of triticale. The WAH means weeks after heading and 150 kg/h a, 225 kg/ha, 300 kg/ha represents seeding rate per hectare. (TIF) [file pone.0313942.s008.tif]

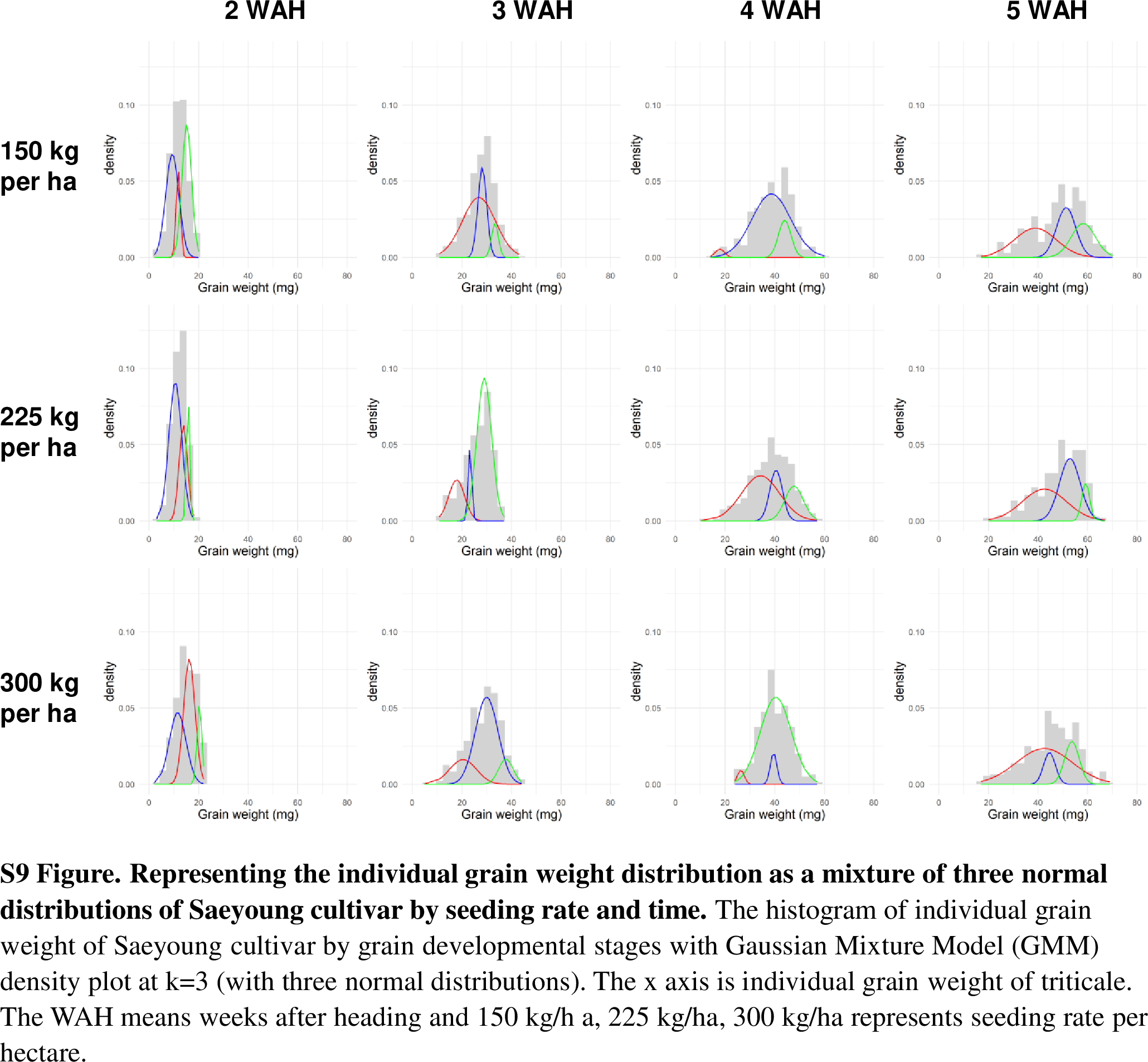

Supplement: S9 Fig — The histogram of individual grain weight of Saeyoung cultivar by grain developmental stages with Gaussian Mixture Model (GMM) density plot at k = 3 (with three normal distributions). The x axis is individual grain weight of triticale. The WAH means weeks after heading and 150 kg/h a, 225 kg/ha, 300 kg/ha represents seeding rate per hectare. (TIF) [file pone.0313942.s009.tif]
