# Supplementary material for: Deciphering individual triticale grain weight patterns: A gaussian mixture model approach
Source: PLoS One. 2024 Nov 26;19(11):e0313942. doi: 10.1371/journal.pone.0313942 (PMC11594513; doi:10.1371/journal.pone.0313942)
Supplement: S1 Table — The temperature and precipitation data from the nearest meteorological observation station to the experimental site, sourced from the Korea Meteorological Administration from September 2022 to August 2023. (DOCX) [file pone.0313942.s010.docx]

**S1 Table.** **The temperature and precipitation data for experimental site.** The temperature and precipitation data from the nearest meteorological observation station to the experimental site, sourced from the Korea Meteorological Administration from September 2022 to August 2023.

|  | Temperature (℃) | | | Average Precipitation (mm) |
| --- | --- | --- | --- | --- |
|  | Mean | Max | Min |  |
| Sep, 2022 | 20.0 | 30.8 | 8.6 | 204.5 |
| Oct, 2022 | 12.3 | 26.8 | 0.2 | 146.3 |
| Nov, 2022 | 8.0 | 22.4 | -9.4 | 88.7 |
| Dec, 2022 | -4.9 | 10.1 | -16.8 | 12.0 |
| Jan, 2023 | -3.7 | 13.2 | -20.3 | 49.8 |
| Feb, 2023 | 0.6 | 12.9 | -8.9 | 0.5 |
| Mar, 2023 | 8.3 | 26.4 | -4.7 | 14.4 |
| Apr, 2023 | 12.7 | 28.7 | -0.7 | 94.6 |
| May, 2023 | 18.0 | 30.8 | 4.5 | 158.7 |
| Jun, 2023 | 22.0 | 33 | 13.8 | 218.4 |
| Jul, 2023 | 25.6 | 33.5 | 18.7 | 355.6 |
| Aug, 2023 | 20.0 | 30.8 | 8.6 | 255.9 |
