# Supplementary material for: Deciphering individual triticale grain weight patterns: A gaussian mixture model approach
Source: PLoS One. 2024 Nov 26;19(11):e0313942. doi: 10.1371/journal.pone.0313942 (PMC11594513; doi:10.1371/journal.pone.0313942)
Supplement: S2 Table — Shapiro-Wilk normality test on individual triticale (X Triticosecale Wittmack) grain weight. (DOCX) [file pone.0313942.s011.docx]

**S2 Table.** **Normality test for each distribution.** Shapiro-Wilk normality test on individual triticale (X *Triticosecale* Wittmack) grain weight.

| Variety | Sampling | Seeding rate | Grain weight ^a^ | Min-Max scaled  grain weight |
| --- | --- | --- | --- | --- |
| GW ^b^ | 2 WAH ^c^ | 15.0kg/10a | *** | *** |
|  |  | 22.5kg/10a | *** | *** |
|  |  | 30.0kg/10a | *** | *** |
|  | 3 WAH | 15.0kg/10a | *** | *** |
|  |  | 22.5kg/10a | * | *** |
|  |  | 30.0kg/10a | *** | *** |
|  | 4 WAH | 15.0kg/10a | ** | *** |
|  |  | 22.5kg/10a | *** | *** |
|  |  | 30.0kg/10a | *** | *** |
|  | 5 WAH | 15.0kg/10a | *** | *** |
|  |  | 22.5kg/10a | *** | *** |
|  |  | 30.0kg/10a | *** | *** |
| MP | 2 WAH | 15.0kg/10a | *** | *** |
|  |  | 22.5kg/10a | *** | *** |
|  |  | 30.0kg/10a | *** | *** |
|  | 3 WAH | 15.0kg/10a | *** | *** |
|  |  | 22.5kg/10a | 0.243 | *** |
|  |  | 30.0kg/10a | 0.285 | *** |
|  | 4 WAH | 15.0kg/10a | *** | *** |
|  |  | 22.5kg/10a | *** | *** |
|  |  | 30.0kg/10a | *** | *** |
|  | 5 WAH | 15.0kg/10a | *** | *** |
|  |  | 22.5kg/10a | *** | *** |
|  |  | 30.0kg/10a | *** | *** |
| SY | 2 WAH | 15.0kg/10a | *** | *** |
|  |  | 22.5kg/10a | *** | *** |
|  |  | 30.0kg/10a | *** | *** |
|  | 3 WAH | 15.0kg/10a | *** | *** |
|  |  | 22.5kg/10a | *** | *** |
|  |  | 30.0kg/10a | ** | *** |
|  | 4 WAH | 15.0kg/10a | *** | *** |
|  |  | 22.5kg/10a | ** | *** |
|  |  | 30.0kg/10a | 0.160 | *** |
|  | 5 WAH | 15.0kg/10a | *** | *** |
|  |  | 22.5kg/10a | *** | *** |
|  |  | 30.0kg/10a | *** | *** |

^a^ Each value was calculated from the average of individual grain weight from the heaviest five heads out of a total of 60 samples. * represents P < 0.05, ** P < 0.01, *** P < 0.001 by Shapiro-Wilk normality test.

^b^ GW: Gwangyoung, MP: Minpung, SY: Saeyoung cultivar

^c^ WAH stands for Weeks After Heading, indicating the timing of sampling after the heading date.
