# Supplementary material for: Deciphering individual triticale grain weight patterns: A gaussian mixture model approach
Source: PLoS One. 2024 Nov 26;19(11):e0313942. doi: 10.1371/journal.pone.0313942 (PMC11594513; doi:10.1371/journal.pone.0313942)
Supplement: S3 Table — The log-likelihood values from the GMM analysis for the three cultivars harvested in Pocheon, Gyeonggi Province, South Korea in 2023. The k = 1 means the assumption that the sample distribution is made up of one normal distribution, k = 2 assumes two normal distributions, and k = 3 assumes three normal distributions. (DOCX) [file pone.0313942.s012.docx]

|  | Seeding rate | GW | | | MP | | | SY | | |
| --- | --- | --- | --- | --- | --- | --- | --- | --- | --- | --- |
|  |  | k=1 | k=2 | k=3 | k=1 | k=2 | k=3 | k=1 | k=2 | k=3 |
| 2WAH^a^ | 150 kg/ha | -1032 | -1023 | -1019 | -1060 | -1042 | -1034 | -1081 | -1075 | -1073 |
|  | 225 kg/ha | -695 | -684 | -683 | -899 | -889 | -887 | -786 | -772 | -770 |
|  | 300 kg/ha | -827 | -788 | -786 | -935 | -912 | -911 | -900 | -892 | -886 |
| 3WAH | 150 kg/ha | -852 | -840 | -839 | -1108 | **-1097** | -1098 | -1230 | -1221 | -1217 |
|  | 225 kg/ha | -1032 | -1030 | -1024 | -1010 | -1007 | -1006 | -1066 | -1040 | -1034 |
|  | 300 kg/ha | -866 | -859 | -853 | -1235 | -1233 | -1232 | -1159 | -1153 | -1152 |
| 4WAH | 150 kg/ha | -1041 | -1032 | -1030 | -1315 | -1292 | -1291 | -1447 | -1436 | -1433 |
|  | 225 kg/ha | -1167 | -1153 | -1148 | -1195 | -1171 | -1169 | -1038 | -1033 | -1031 |
|  | 300 kg/ha | -994 | -975 | -972 | -1151 | -1136 | -1135 | -950 | -946 | -945 |
| 5WAH | 150 kg/ha | -859 | -848 | -839 | -1297 | -1278 | -1278 | -1359 | -1341 | -1340 |
|  | 225 kg/ha | -1011 | -994 | -991 | -1329 | -1316 | -1314 | -1383 | -1354 | -1350 |
|  | 300 kg/ha | -983 | -944 | -939 | -1515 | -1501 | -1498 | -1479 | -1467 | -1464 |

**S3 Table. Representing the log-likelihood values by grain developmental stages of Gwangyoung (GW), Minpung (MP), and Saeyoung (SY) cultivar.** The log-likelihood values from the GMM analysis for the three cultivars harvested in Pocheon, Gyeonggi Province, South Korea in 2023. The k=1 means the assumption that the sample distribution is made up of one normal distribution, k=2 assumes two normal distributions, and k=3 assumes three normal distributions.

a) WAH: Weeks After Heading
