# Supplementary material for: Deciphering individual triticale grain weight patterns: A gaussian mixture model approach
Source: PLoS One. 2024 Nov 26;19(11):e0313942. doi: 10.1371/journal.pone.0313942 (PMC11594513; doi:10.1371/journal.pone.0313942)
Supplement: Attachment — Submitted filename: Response to Reviewers.docx [file pone.0313942.s014.docx]

Response to Reviewers

PLOS ONE

Academic Editor

Dear Dr. Soltani-Gerdefaramarzi,

Thank you for the opportunity to revise our manuscript and for the constructive feedback provided by editor and the reviewers. We have carefully addressed each point raised and have made the necessary revisions to enhance the quality and clarity of our manuscript.

We have reviewed the formatting guidelines provided in the PLOS ONE style templates (PDF file) and the submission guidelines at https://journals.plos.org/plosone/s/submission-guidelines#loc-laboratory-protocols. Accordingly, we have updated the title and headings to ensure they meet the capitalization rules and have added a Conclusion section while revising the ending of the Discussion section for coherence.

Additionally, we have corrected errors in the table values and used the formatting tools at https://pacev2.apexcovantage.com/ to ensure that the figures meet the required format and have renamed the files appropriately.

We have revised our data availability statement in line with PLOS ONE's Data Policy. We have made the data available in accordance with PLOS ONE's Data Availability policy. We have included the individual grain weight data and the code used for GMM analysis in the supporting information section.

The instances of "data not shown" in our manuscript have been addressed. We have added the relevant data to Supplementary Table 3 to ensure compliance with PLOS ONE's data sharing requirements. All relevant data are now accessible either within the paper or through the supplementary files.

We believe these revisions address the reviewers' concerns and improve the overall quality of our manuscript. Thank you for considering our revised submission. We look forward to your positive response.

Kind regards,

Prof. Dr. Wook Kim

Reviewer 1.

1. the wrtitng style of the paper should be checked because there are many unclear sentences and long sentences in the paper.

We have revised the sentences throughout the paper to improve clarity and shorten overly long sentences.

Line 62 – 65.

Before: The triticale (× *Triticosecal*e Wittmack), which developed from the hybridization of wheat (*Triticum* spp.) and rye (*Secale cereale* (L.) M.Bieb., Poaceae), is primarily produced in Europe, and its production has been steadily increasing each year [18, 19].

After: The triticale (× *Triticosecal*e Wittmack), which developed from the hybridization of wheat (*Triticum* spp.) and rye (*Secale cereale* (L.) M.Bieb., Poaceae). It is primarily produced in Europe, and its production has been steadily increasing each year due to its value as a feed grain [18, 19].

Line 128 – 130.

Before: The ‘normalmixEM’ function in ‘mixtools’ package was used for calculating log-likelihood Gausiaan mixture model (GMM) at k = 2, and 3. For k = 1, the log-likelihood was obtained by calculating the fit of a simple normal distribution.

After: The ‘normalmixEM’ function in ‘mixtools’ package (version 2.0.0) was used to calculating log-likelihood Gausiaan mixture model (GMM) at k = 2, and 3. For k = 1, the log-likelihood was obtained by fitting a simple normal distribution.

Line 341 - 347.

Before: Moreover, in Table 5, the MP cultivar shows the highest average grain weight and the proportion of distributions with a high average at 5 WAH, but according to GMM analysis results, GW and SY exhibit a smaller standard deviation in the distribution with the higher mean compared to MP, indicating a more uniformity. This suggests that distribution characteristics need to be considered in selection or comparisons between cultivars.

After: Moreover, in Table 5, the MP cultivar shows the highest average grain weight and proportion of distributions with a high mean at 5 WAH. However, in Table 5, GW and SY exhibit a smaller standard deviation in the distribution with the higher mean compared to MP, indicating a more uniformity. This suggests that distribution characteristics need to be considered in selection or comparisons between cultivars as perspective of uniformity. Traditional representative values, such as the mean, are often inadequate for comparing and analyzing distributions that deviate from normality, such as bimodal distributions. This demonstrates that GMM can be effectively used for comparing and analyzing such distributions.

Additionally, we have modified other long and unclear sentences and words throughout the manuscript like removing repetitive phrases such as "as mentioned".

2. There is no caption for the figures under the figures.

According to the journal format, figure captions should not be placed under the figures but in the main text. We have added the captions to the main text.

Line 177 – 180, Line 203 – 222

3. Version of R along with RStudio should be specify separately in the Statistical section.
specify the code that is used for the analysis.

Line 120 to 135.

We have added the versions of R, RStudio, and the packages used, along with the commands used in the analysis, to the Materials and method section. Moreover, we have included the R studio code used for GMM analysis in the supporting information section.

4. The sampling was begun at heading stage, while at this stage there is no filled grain to be weighted. Also, it is not clear for me what is the aim for sampling over time? I could not find its application on the suggested distribution!
specify why different seed density for cultivation has been applied in the experiment.
Is there any differences among your treatment, seed density and cultivars?

the discussion is somewhat weak, and it might be better to rewrite it focusing on the application of the suggested method in the paper.

The goals of this experiment were:

To determine if the grain weight distribution of triticale follows a normal distribution. If not, what distribution does it follow?

To identify whether the grain weight distribution of triticale has a specific form and quantifying it. Moreover, does the form generally remain the same while exhibiting differences depending on the cultivar?

Sampling over time was conducted to verify whether the distribution form changes over time, addressing the experimental objective of identifying if the distribution form has a specific shape. Through min-max scaling in the supplementary figure, we confirmed that samples at 2 WAH and 3 WAH, which are difficult to observe in the histogram, also show a left-skewed distribution with a long left tail.

Additionally, the GMM analysis results indicated that all three cultivars exhibited a specific left-skewed form. While maintaining this specific form, differences between the three cultivars were also observed.

We apologize for the lack of details regarding the experimental goals. We have revised the introduction and discussion sections to include this information and added information to the discussion section on how the results can be applied. (Introduction section (Line 67 -78), Discussion section (Line 341 - 347, Line 354 - 361), and Conclusion section (Line 391 - 403).

Moreover, we have added information to the discussion section on how the results can be applied.

5. Do you think that the number of sampled grains is enough to suggest a new distribution for grain weight?

Although the number of heads sampled per treatment (five) might seem small, we believe it is sufficient to suggest a new distribution due to the consistency of the results observed.

Thank you for your valuable feedback. We apologize for any confusion caused by the lack of clarity regarding the objectives of the study.

Reviewer 2.

First, we would like to express our gratitude for your valuable comments.

We have addressed the points you raised as follows:

1. The quality of figures could be better.

We have added borders to the figures to make them clearer and have included the means of each distribution and the proportion that each distribution contributes to the overall distribution. This information has been added to the figure legend (Line 203–222).

2. No powerful literature review can be observed in the introduction. Similar research should be reviewed, and the novelty of your research compared to them should be mentioned in the text.

Upon review, we realized the last paragraph of introduction section was indeed lacking. We have added relevant studies and expanded on the explanation of GMM and the objectives of this study. (Line 67 – 78)

Once again, thank you for your thorough review.

Best regards,
