# Supplementary material for: Deciphering individual triticale grain weight patterns: A gaussian mixture model approach
Source: PLoS One. 2024 Nov 26;19(11):e0313942. doi: 10.1371/journal.pone.0313942 (PMC11594513; doi:10.1371/journal.pone.0313942)
Supplement: Attachment — Submitted filename: Response to Reviewer 3.docx [file pone.0313942.s015.docx]

Reviewer 3.

Comments 1: The manuscript “Deciphering Individual Triticale Grain Weight Patterns: A Gaussian Mixture Model Approach” present a novel interpretation of weight distribution and structure of individuals grains in triticale (*Triticosecale* Wittmack) from the perspective of mixture Gaussian distribution. The descriptions are presented appropriately and the design factors are included properly. There are however, few concerns that would like to address in the following that could be explained better to be clarified to those readers interested in this topic:

Answer 1: The analysis for this issue is currently in progress. Initially, the grains were measured without considering a specific distribution shape, as the distribution form had not been identified at that time. Currently, we are analyzing this in another sampling dataset. The triticale cultivars used in this analysis mainly produced grains in the 1st and 2nd florets, similar to rye, and occasionally in the 3rd floret. Interestingly, during the analysis of the distribution of grains developed in the 1st and 2nd florets, we observed that each showed a left-skewed distribution.

However, since this analysis is still in progress using a different dataset, it is difficult to mention this in the main text (as per PLOS ONE's guidelines, "data not shown" is not accepted). Additionally, because this is from a different dataset, we believe it would be difficult to include it in the supplementary materials as well.

Therefore, we have added a brief note on the ongoing analysi on line 373 to 374.

Comments 2: I am not sure whether the individual weights (proportion of individual grain memberships) inferred from mixture models specially at k values 2 or more could be derived from the candidate model(s). If yes, then what would be the consequence if some individuals could not be classified into specific distribution? Are such individuals being wrongly sampled as they are potential admixtures? Please explain how the proposed methods could deal with these admixed individuals.

Answer 2: In GMM, all data points always have a probability of belonging to at least one distribution. While there is a possibility that some data may be assigned to the wrong distribution, this is inherent to GMM’s probabilistic nature of assigning data.

Line 108 to 111, We have added an explanation of how the data is assigned in the GMM.

We believe that the primary issue affecting the reliability of the analysis is the presence of outliers. Therefore, before analyzing the dataset, we conducted outlier removal, and since we divided the samples present in this dataset, we consider the analysis to be reliable.

Comments 3: As mentioned in the manuscript, the number of individuals analyzed are varied among different combinations (ranged from 236 to 408). There must be some problems in defining the bin widths in the histogram of the individual weight values and comparison between different combinations of time*seed rate.

Answer 3: In the case of the histogram, the command was used to divide the data into 30 equal bins. In line 134 to 135, we have added that histogram information

As you mentioned, differences in shape could arise due to the different sample sizes; however, there are min-max normalized density graphs presented in Supplementary Figures 1-3, which help to identify these differences. This information has been added to the main text, line 361 to 362 in discussion section.

Thank you once again for your valuable review comments.
